# Valedictory Address before the Aesculapian Society, at Charleston, Ill., Nov. 28th, 1855

**Published:** 1856-03

**Authors:** Thos. D. Washburn

**Affiliations:** Lawrenceville, Ill.


### THE NORTH-WESTERN

### MEDICAL AND SURGICAL JOURNAL.

(NEW SERIES.)

VOL. V. MARCH, 1856. NO. 3

### ORIGINAL COMMUNICATIONS.

ARTICLE I.—*Valedictory Address before the Aesculapian Society, at Charleston, Ill., Nov. 28th*, 1855. By THOS. D. WASHBURN, M.D., of Lawrenceville, Ill.

It has been customary and the Constitution makes it obligatory for the presiding officer, to close his official term with an address to the society.

In obedience to this requisition, I appear before you, not in the capacity of a public speaker so much as an humble individual of a noble profession, to give utterance to those sentiments which I trust will find a cordial response in every bosom.

I shall not invite your attention to the subtilties of our profession; nor shall I lead you into the mazy labyrinths of medical science—its theories and dogmas I shall studiously avoid.

I leave these matters to those who have more elegant leisure: to the fathers of the profession, who have more ease and affluence, by their genius and industry, and retiring on their laurels, are ready to vindicate and elucidate abstruse views or favorite theories.

I shall only aim to present a few *practical* items, suggested, mainly, by our *present position*, and the relative duties which grow out of it.

Crowded as we have been the past season, with endless business, and worn down with watching and fatigue, you can but expect from me a meagre, detached, desultory, and almost agueish effort.

It affords me great pleasure, however, to meet so many of my brethren and fellow citizens, to enjoy that social intercourse, and interchange of sentiment and sympathy, which has a meaning and intensity among ourselves, that no other brotherhood, united by natural or artificial ties, can either approximate or appreciate.

From the earliest ages, so far back as history gives us any record, some of the human family have been devoted to the care of the sick and the cure of disease.

Prominent and more exclusive at first, were an order of men set apart to minister in *divine* things, and teach the people the worship of a true or false God. For awhile medicine was associated with these, but in the course of human events they became separate, and from that time to this, centuries ago, the *Physician* has had a name and a place distinct and apart from the Priest! Nor need we be ashamed of our origin or association, for with them, the priests, were the highest attainments in knowledge, science, and literature.

Since these bonds were severed, the Physician has been a name the people delighted to honor. He has been made the companion of Kings and learned men, and while he entered alike the palace and the hovel, he has received the most flattering homage and the most considerate regard.

Such has been the condition of the physician in most of the kingdoms of the Old World, and such it continues to be at the present day; true, high professional attainments are required, and the student must consume time and money and labor, before he can pass the rigid ordeal that confers a degree, but having accomplished this, he stands upon the broad platform of professional competency, and takes position among the wise and great who have preceded him. The sovereignty of law protects him in his business and shuts out those not properly qualified. Every avenue of professional distinction is open before him; while the prospect of affluence and reputation stimulate him to that attention to business which insures success, without being annoyed by the intrigues of base pretenders, or outstripped by ignorance and conniving. Such is a hasty glance at the position of the profession in foreign countries. How is it in our own?

At first the older States, under the influence of home education and example, were pleased to restrict the practice of medicine and surgery to those only, who could sustain a fair examination, or present the proper credentials from some well regulated and chartered Institution. But Young America, not willing to be bound to her mother's apron string! and having a little hereditary distaste for anything that curtailed the greatest liberty, abrogated most of these restrictions,—threw the doors wide open to the quack, the knave, and the ignoramus, invited the learned and unlearned to meet on a common level, and forced science and acquired skill to cope with sophistry, unprincipled cunning, and barefaced impudence; giving the untaught multitude the chances of life or death, guided mainly by human instinct, for that judgment which is formed by mere external appearances is as fallacious as the dreams of boyhood, and deserves no higher name than instinct.

Even at the present time, while the regular practitioners are endeavoring to raise the standard of professional excellence, protracting the course of study, and enforcing more rigid scrutiny in their examinations, charlatanry addresses every phase of society with brazen assurance, and seizing on some popular prejudice, or adopting some favorite appellation, without science, skill, or knowledge, they engross public favor, and make their way to the prejudices and purses of their patrons, secure the influence of good men and honored citizens, while modest, but genuine merit, too often goes unrewarded.

I know there is a popular notion abroad, that "every thing finds its proper level," and so it does, as a general thing, in older communities among intelligent masses, but any one that will examine the history of the West, and scan its rise and progress for the last twenty-five years, will abandon this idea. A country made up of a most heterogeneous compound of all tongues, and nations, and languages, learned and unlearned, many grossly ignorant, and thousands wantonly superstitious, the most credulous and the most skeptical, they become the fit tools for demagogues, political, medical, and religious, blown about by every wind of doctrine, they were as unstable as water, and ripe subjects for quackery and empiricism.

But a brighter day is dawning—with a rich soil, and a rapidly increasing population, with eminent commercial facilities, a better class of emigration, a speedy transit for travel or produce, a high tone of morals, a higher standard of education, and the vast diffusion of knowledge among the masses. The times are passing by, when a barrel of whisky will place a man in the halls of legislation, or the tongue of medical pretention assume the dignity and position of science.

But to define the relative position of the two great classes of medical practitioners of the present day, allow me to draw a parallel by presenting a medical student from each—take a *medium* case if you please—and look *first* on this side and *then* on that!

At the outset, the student of medical science selects a physician of good reputation, with the best library and the most facilities for acquiring a knowledge of his profession. He takes up the subject of anatomy, and one by one he gains a knowledge of the bones, their size, shape, position, uses, &c. He clothes them with muscles, and ascertains the situation and relation of the nerves, the arteries, and the veins; with still deeper interest he studies the internal viscera, the vital organs, and learns their character, use, and locality.

He then applies himself to physiology, learns the laws of life, the functions of the different glands and organs of the body, their dependence and connexion with each other, and their relation to external objects. Having found their condition in a *healthy* state, he next seeks pathology, and learns the condition of these various organs in a *diseased* state, a knowledge of which is highly important, for the successful treatment of disease.

Chemistry comes in here to solve many of the processes and operations which he will witness, and he seizes with avidity upon its teachings, and makes himself master of a thousand processes which before were inexplicable. He acquires a knowledge of the elements of matter, their action and reaction on each other, and while it is brought to bear on the living organism, his acquaintance with physiology will not allow him to overlook the great *vital principle* which overrules and controls all other agents in the human system. Dismissing chemistry, he now enters upon the study of materia-medica and therapeutics, and here he dwells long and laboriously to master the various agencies which are presented for controlling and modifying disease. Their nature and combination must be known, to act efficiently against the arch enemy of mankind, disease and death.

Having laid the foundation, he begins now to build up the superstructure, by coning over the best living and dead authorities on theory and practice, surgery, midwifery, diseases of women and children, &c., &c.; his mind, disciplined by study and reflection, his judgment matured, his memory quickened, he wanders from author to author, devouring, digesting, and elaborating knowledge; interested and delighted, he casts about for more food to supply the never-satisfied mind; he makes his way to the city, and enters the Hospital, the Infirmary, and the Lecture Room, and draws huge draughts from the fountains of living knowledge; his erroneous views are eradicated, his various acquirements systematized, his professional zeal excited, and he leaves his *alma mater*, furnished and prepared to enter upon his glorious mission.

But the student of medical quackery, pregnant with the idea that greatness sleeps within him, and the world is suffering for want of its speedy development, bursts his shell of obscurity, picks up a Thompsonian practice—reads a few water-cure journals, loafs a few weeks in an empiric's office, is told that lobelia, red-pepper, dog-wood, and pleurisy root were almost the sole agencies used by the ancients to combat disease, and that the Indians are the great depository of all the medical wisdom of past ages; that all minerals are deadly poisons, and that the whole vegetable creation is perfectly harmless. Thus equipped, he starts out on his mission of charlatanry with brazen arrogance, regardless of moral as well as physical laws, he puts forth his assumed title and malicious hand bill, (the finger-board to death,) and boldly commences the treatment of the most complicated diseases; ignorant of the human system, and ignorant of the agencies with which he operates. Case after case proves fatal, and yet he still persists, till calloused in hardihood, he can number scores that reason and conscience thunder in his ears have fallen victims to his slaughtering treatment.

But the prefix of "Doctor" protects him from violent hands. Ashamed to yield, he removes his residence to some other field and renews the attack with redoubled vigor. He assures the public, that he has examined both systems, that the old one is crumbling to decay, but *his* system is the only one rational and perfect, that he never lost a patient, and no one need die that will send for him in proper season; in fact, there is no end to his arrogant assumption, and he bewilders some and converts others to his theories and vagaries.

Such is an imperfect but true sketch of many we have to contend with in our professional career, bereft of all the rules of propriety, they seek out our patients in our absence, undermine our influence, work upon the fears and hopes of the feeble and diseased, till they succumb occasionally to their infernal devices, and perhaps pay the penalty with their lives.

Such is the position of regular medicine in regard to quackery.

And why not more successful in sweeping it into chaos and oblivion? Because we have no laws to prevent quacks assuming our legitimate name, nor the community any law to protect themselves from their barbarous and cruel invasion.

We have laws to protect men in the enjoyment of their property, but no laws to protect them in the enjoyment of their health, even when Providence is disposed to grant them such a boon. We have laws to guard against swindling and fraud, against poisoned meats and unwholesome drinks, against moral and criminal outrage, but nothing on the statute to prevent one destroying health, sapping and undermining the system by slow but certain poison, and gradually sinking them into an untimely grave, provided it is accomplished medically.

Myriad quacks may swarm and cover the land, they may swindle and deceive the public, and rob them of their houses and lands, their gold and silver, and their cattle, by incompetent knowledge of medical science, but it is borne submissively, while the poor destitute laborer, whose necessities force him to snatch a loaf of bread, or steal a few farthings to procure the same, is hurried off to the penitentiary.

The law demands requisite qualifications in those who instruct our youth in the simple elements of education; while the variest simpleton, who can neither read or write, is allowed to drug and poison whole families and communities.

Lawyers must have a good knowledge of their profession, and be able to sustain a fair examination before they can enter upon business and appear at the bar; but physicians are held accountable at no bar, not even at the bar of public opinion. Alas! how awful and solemn will be the acconnt that many will have to render at the Bar of the Almighty! It is painful to follow this theme and realise how callous public sentiment is to a just appreciation of right in regard to these matters. But the *evil* exists, and the question arises, what shall be done to remove it? The language of denunciation is not the proper remedy. It must be reasoned down, lectured down, wrote down, and legislated down.

Let us canvass the subject, and not fear to meet any of the prejudicies which may exist against our own system. Let us publish and scatter broadcast proper views on the subject of medicine. While the press teems with nostrums and specifics, and quack advertisements of the most rediculous and absurd character, let us devote at least one column in every family newspaper to subjects and matters connected with medical science. If we occupy the right position, why not defend it? Let us no longer hide our light under a bushel. Throw your technical and Latin terms away when you talk to the popular mind; you will be none the less respected, and they are not essential to professional salvation. Let us insist that physiology be more thoroughly and extensively taught in our schools, take the trouble to go in and examine the classes yourselves, and see that it is made interesting, not repulsive.

Demand of our State legislature that all nostrums, specifics, and patents be labeled with the proper English names of their ingredients. Let a petition to this effect be circulated, and if you will use half your influence, half the people of the State will sign it, and it will become a law, and then Horatio's trade is gone.

Bring before the American Medical Association the necessity and propriety of Congress repealing all laws recognizing the existence of patents in anything connected with the profession, medical or surgical. It is an infringement on our rights, derogatory to our character, and holding out a false impression to the world. Let it be blotted from the statute book.

Our duty to *pretenders* then, is, while we come out and be separate, stand aloof and touch not the unclean thing, to attack not so much the *men* as the *system;* true it would be well, from time to time, to publish instances of gross misconduct and flagrant violations of all science and common sense, which are constantly occurring under our own observation; perhaps in some cases give individual examples of the history, acquirements, and habits of notorious quacks, but in the main, let us direct our batteries to the *measures* and *principles* which they advocate, as well as the general incompetency of those who attempt to carry them out.

It is good to be zealously affected in a good cause. Many of the evils, gentlemen, which we have to contend with, have arisen from our own negligence or false modesty. Let us arouse from our lethargy and proclaim upon the house tops, if need be, the dangers of the hydra-headed system of quackery. Expose its cloven foot, and let the light of truth shine with such dazling rays upon its treachery and baseness, that the most unskilled and common mind may behold its deformity, then *one* shall chase a thousand, and *two* put ten thousand to flight.

But we have duties which we owe to ourselves and one another, which should not be overlooked or neglected. Our excellent code of medical ethics points out the great landmarks which should guide us in our various relations. Let it be studied, till we can master both the letter and the spirit which is found there; but there are other items not specified which must not be disregarded.

We owe it to ourselves to keep faithful watch that incompetence enter not our own borders. We should keep posted up with the medical progress of the age—to do this, requires not only that we *take*, but that we *read* at least one or two medical journals. We should be fully equipped with the various therapeutic agents; it is a shame to be constantly substituting one remedy for another—it is an outrage on our patient and an injury to ourselves to indulge in this liberty to any extent.

Brethren, it is the great opprobrium of the profession that we do not "Love one another" any too well. I acknowledge it with shame.

The low, selfish, sordid principles which rule among the ignorant and vicious; the querulous competition which is exhibited by picayune tradesmen, shop boys, and hucksters, has no business or right in our ranks. The trite old adage that "two of a trade do not agree," ought never to have been breathed in allusion to ourselves.

I know that conflicting interests will arise. Our habits and temperaments are so diverse, our prejudices so strong that two cannot always walk together, but often the most trivial matter has separated those who should be friends. We are, as a profession, to sensitive in regard to points of etiquette, dignity, and reputation, and, if the truth was told, we shall find that the Almighty Dollar plays no small figure in these defections.

The elder, grown old in the affections of his faithful patrons, and somewhat indolent and careless from his continued labors and increasing cares, is taken off his guard, and speaks harshly at times when some youthful aspirant locates near the old homestead, and begins to encroach on what he considers his legitmate domain; the younger, poor but spirited, stands on his reserved rights, and stung to the quick by some unkind word or treatment, at once becomes alienated. These things ought not to be. Fortunately these social gatherings which we are permitted to enjoy to-day, the influence of associations, the workings of these local societies, which are steadily increasing, will do very much to remedy these evils, we trust, effect a radical cure.

Changing the topic, (for family failings ought not to be exposed to the public gaze,) it becomes us, as physicians, to forewarn and thus forearm community and individuals against danger and disease; to instruct them publicly and privately in regard to preventing the access of disease, the influence of sudden changes in the temperature, and the necessity of adapting their clothing to the same; the influence of miasma—the depressing nature of fear and panic—the genial influence of bathing, and the counteracting agency of cleanliness in warding off contagion, cholera, &c. These, and a thousand other individual and general causes, which the true physician is able to detect, demand his attention and should receive his notice.

But above all, we owe it to ourselves and community, to be devoted to our profession, to yield to no depression or fears, or anxieties, to no temptations which poverty may urge, or want of appreciation suggest; but labor and study to meet every exigency, and with an eye steadily fixed on ultimate success, indifferent to calumny and unbiased by praise, patient, but vigalant, ever at our post, let us shun the *evil* and the *good* pursue, for

> "The evil men do lives after them,
> The good is oft interred with their bodies."

Let professional excellence be our "*motto*," and if the present do not appreciate it, perhaps posterity may.

Our obligations to the learned professions are of a mutual character; we stand on a common platform, our literary acquirements are near the same, we grew up school-mates, class-mates, and room-mates, from the common school to the academy, and to the close of our collegiate course, when we separate and each pursues the profession of his choice. To the Bible we owe the high position we occupy as a nation, and those who faithfully expound its doctrines, and preach the pure gospel of revelation, demand our love, respect, and admiration; we should sustain them by our influence and hearty co-operation.

The law, too, is a profession as indispensable to keep the machinery of society in proper order and motion, as either or all others. They are our compeers, and from them, to a large extent, we look for our highest state and national officers. They, more than any *one* class, control our elections, our legislation, and our judicial decisions.

Each profession has its own peculiar sphere and is as essential to the body politic as the hand, or arm, or foot is essential to the physical frame.

There is a community of interest, and there should be a community of feeling, existing among all the learned classes of society. To a large degree they create the tone of society on the moral, social, political, and religious questions of the day; and posterity will hold them responsible for their healthy or diseased condition. As members of a sister profession, let us frankly and cheerfully concede to them all their personal rights and professional privileges, and as "love begets love," we may fondly hope the day is not far distant, when we shall have their combined influence in elevating our position as a profession; while they shall mete out justice to those that have not enjoyed that freedom and protection which their purity, intelligence, and genius justly deserve.

In a word, the professional classes are entrusted, to a great extent, with the interests of philanthrophy and education, with the political and religious bias of the people, with the moral and social position of community. Thus affiliated, we ought cordially to assist in bearing one another's burdens, and he who boldly denounces or publicly detracts from the merits of either, as a class, proves recreant to the best instinct of his nature, tramples upon the public weal, and justly deserves the finger of scorn and contempt.

But, my friends, in conclusion, we have no reason to despair, on the contrary, we have very much to encourage us; like the century plant, we are of slow but certain growth, we are not anxious to boast on empherial existence; ours has been the growth of ages and centuries. We take no pride in being considered "fast" men, and we have no more affinity for one idea systems than we have for alligators. We take the liberty to cull and appropriate from all systems, whatever we can find useful or meritorious. We intend to have Nature minister to our wants and necessities, whether the agent comes from the *vegetable*, *mineral*, or *animal* kingdom. These principles are inherent in our system of medical science, and as such, bound to be progressive. We are striving for *perfection*, yet we seem never likely to reach *maturity*.

We are like the apostle Peter after his vision, not disposed to call that unclean or worthless that the God of Nature has placed in our hands. I said we have no reason to despair; neither the past or present fills our minds with doubt or intimates any uncertainty of ultimate success. We have names high on the list of fame as men of literature, science, and philanthropy. While we are proud of the bright galaxy which shine on the historic page of other lands, we boast of not a few in our own land, and shall never cease to cherish the memory of a Rush, a Physic, and a Parish, a Dewees, a Gibson, and a Drake. Even at the present time, on the present stage of action, we could enumerate a host as devoted, as brilliant, and as noble as any of their illustrious predecessors, and many not yet in the zenith of their fame. Nor should I pass in silence our National Association, which is rapidly giving our profession a *national* character; developing our medical resources, creating harmony of action and feeling; elevating and purifying the whole medical mass. The volume of transactions, which it annually sends forth, will bear the most rigid scrutiny as a literary and scientific work.

The topography of different localities, with the character of the prevalent diseases in the same, which is so faithfully recorded here, make it of inestimable value to the junior members of the profession; indeed, reflecting as it does the type of the most distinguished minds among us, combining a freshness and originality which few works possess, it should be deemed an indispensible acquisition to all our libraries.

Gentlemen of the profession, we stand in the outposts of danger. War and pestilence and famine are abroad in the earth. No class of community are as much exposed to the arrows of death, or the heavy hand of disease, as ourselves. Our task is often a sad and melancholy one. On the very threshold of our profession, we begin to battle with disease and death; his thousand hideous forms are ever lurking in our pathway, reminding us that we, too, are mortal. During the past summer and the one preceding, the pestilence has raged with fearful violence in our Southern cities. How many of our devoted band perished like martyrs!

While others can range creation's bounds unfettered, we must be ever at our posts in the hour of peril. The grim monster may stare us in the face, his icy hand may chill our frames and freeze the very citadel of life, no fear must reach our bosom, no doubt disturb our minds ; inch by inch we dispute his claim, but death finally triumphs. We, too, must pay the debt of nature and journey to our long home. If we have learned wisdom from experience, if the solemn lessons, which have so often been administered in our professional career, have not been like water spilt upon the ground, if we have made the acquaintance of the one *great* Physician who offers freely to heal the dreadful maladies of the soul, who can speak *peace* in life's darkest hour, and pluck the sting of death, *then, all is well.* Professional reputation, troops of friends, and thousands of gold are no panacea for the final hour.

"So live, that when thy summons comes to join
The immeasurable caravan, that moves
To the pale realms of shade, where each shall take
His chamber in the silent Halls of death.
Thou go not like the quarry-slave at night,
Scourged to his dungeon, but sustained and soothed
By an unfaltering trust, approach thy grave
Like one who wraps the drapery of his couch
About him, and lies down to pleasant dreams."

